# The Beat

**DOI:** 10.1289/ehp.119-a384b

**Published:** 2011-09-01

**Authors:** Erin Dooley

## U.S. Forest Service Examines Fire Retardant Policy

As fire season in the western United States peaks, the U.S. Forest Service is incorporating public comments into a draft environmental impact statement (DEIS) published earlier this year.^1^ The DEIS was developed in response to a July 2010 ruling by Montana’s Federal District Court that U.S. Forest Service protocols for aerial application of fire retardants to fight wildfires violated the Endangered Species Act. Although aerial application in remote areas is not considered a direct threat to human health—the smoke from wildfires is deemed a greater hazard^2^—it does carry the risk of inadvertent contamination of waterways and traditional food sources. A final EIS is expected by the end of 2011.

**Figure:**
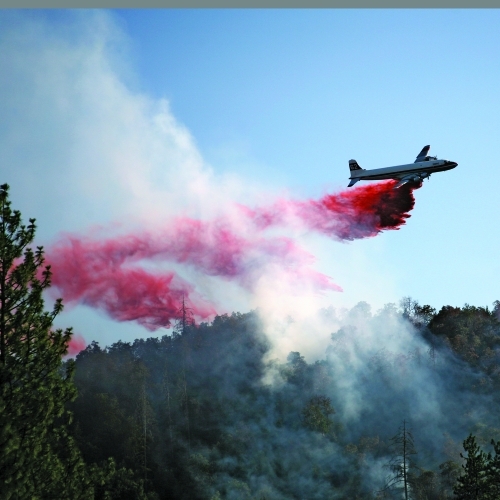
The U.S. Forest Service is assessing the impact of aerial application of fire retardants. Sherri R. Camp/Shutterstock.com

## Teen Hearing Loss Linked to SHS

A new study shows that children aged 12–19 years exposed to secondhand smoke (SHS) were nearly twice as likely as nonexposed teens to experience sensorineural hearing loss, a type of hearing loss typically associated with aging and congenital deafness.^3^ Exposed teenagers performed worse across every sound frequency tested, especially those frequencies vital to understanding speech. Most of the teens with hearing loss were unaware of the deficiency. The researchers point out that although the effects are subtle, they still may be serious enough to impair learning in classroom settings. More than half of all U.S. children are estimated to be exposed to SHS.^4^

**Figure:**
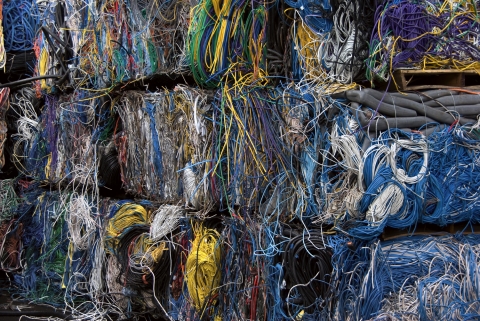
RoHS is expanding to cover all electronic equipment, cables, and spare parts. Huguette Roe/Shutterstock.com

## EU Expands Rules on Electronic Equipment

In July 2011 a new extension to the Restriction on Hazardous Substances (RoHS) directive on electronic equipment entered into force in the European Union.^5^ The extension, which will be phased in by 2019, broadens the scope of the original directive to include all electronic equipment, cables, and spare parts. The extension continues the ban of lead, mercury, cadmium, hexavalent chromium, polybrominated biphenyls, and polybrominated diphenyl ethers from use in electronics, and offers clearer rules for seeking exemptions to the ban. RoHS applies to any business that sells applicable products, subassemblies, or components either directly or indirectly to RoHS countries.

## Casting Nanofiber Nets for Indoor Pollutants

Conventional methods to detect formaldehyde in air can be time-consuming, expensive, and inadequately sensitive. A team of researchers has designed a nanofiber net that, when used as a coating on a device known as a quartz crystal microbalance detector, provides a faster, more sensitive method for measuring low levels of formaldehyde.^6^ The new method uses an electrospinning netting technique to deposit polymide membranes on the microbalance, providing a large surface area and high porosity and adhesive force. The nets also may have uses for detecting viruses and bacteria.

## Exploring the Potential of Frankia

*Frankia* are nitrogen-fixing bacteria that live symbiotically in the roots of actinorhizal plants. A new study shows these bacteria have the genetic capacity to produce products such as antibiotics, herbicides, and anticancer agents.^7^ Researchers used bioinformatic analysis of three strains of *Frankia* to identify dozens of biosynthetic gene clusters—that is, genes used by *Frankia* to manufacture the compounds it needs to survive and thrive. Products have not been observed or characterized for most of these biosynthetic pathways, but this analysis predicts many that are structurally similar to valuable compounds such as vancomycin.

**Figure:**
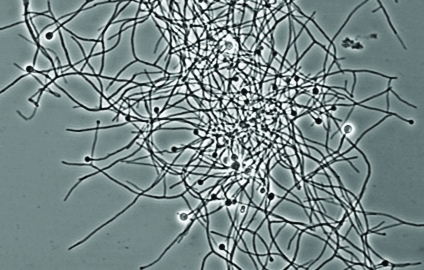
*Frankia* species offer a potential source of useful compounds. Louis S. Tisa/University of New Hampshire
